# Production and characterization of human anti-V3 monoclonal antibodies from the cells of HIV-1 infected Indian donors

**DOI:** 10.1186/1743-422X-9-196

**Published:** 2012-09-12

**Authors:** Raiees Andrabi, Rajesh Kumar, Manju Bala, Ambili Nair, Ashutosh Biswas, Naveet Wig, Pratik Kumar, Rahul Pal, Subrata Sinha, Kalpana Luthra

**Affiliations:** 1 Department of Biochemistry, All India Institute of Medical Sciences (AIIMS), New Delhi, India; 2 Department of medicine, All India Institute of Medical Sciences (AIIMS), New Delhi, India; 3 Medical physics unit IRCH, All India Institute of Medical Sciences (AIIMS), New Delhi, India; 4 Regional STD Teaching Training & Research Centre, Safdarjang Hospital, New Delhi, India; 5 Immunoendocrinolgy lab, National Institute of Immunology, New Delhi, India; 6 National Brain Research Centre (NBRC), Manesar, Haryana, India

**Keywords:** HIV-1, Envelope glycoprotein, Third variable region, Anti-V3 monoclonal antibodies, Viral neutralization

## Abstract

**Background:**

Analysis of human monoclonal antibodies (mAbs) developed from HIV-1 infected donors have enormously contributed to the identification of neutralization sensitive epitopes on the HIV-1 envelope glycoprotein. The third variable region (V3) is a crucial target on gp120, primarily due to its involvement in co-receptor (CXCR4 or CCR5) binding and presence of epitopes recognized by broadly neutralizing antibodies.

**Methods:**

Thirty-three HIV-1 seropositive drug naive patients (18 males and 15 females) within the age range of 20–57 years (median = 33 years) were recruited in this study for mAb production. The mAbs were selected from EBV transformed cultures with conformationally constrained Cholera-toxin-B containing V3C (V3C-CTB) fusion protein. We tested the mAbs for their binding with HIV-1 derived proteins and peptides by ELISA and for neutralization against HIV-1 viruses by TZM-bl assays.

**Results:**

We isolated three anti-V3 mAbs, 277, 903 and 904 from the cells of different individuals. The ELISA binding revealed a subtype-C and subtype-A specific binding of antibody 277 and 903 while mAb 904 exhibited cross reactivity also with subtype-B V3. Epitope mapping of mAbs with overlapping V3 peptides showed exclusive binding to V3 crown. The antibodies displayed high and low neutralizing activity against 2/5 tier 1 and 1/6 tier 2 viruses respectively. Overall, we observed a resistance of the tier 2 viruses to neutralization by the anti-V3 mAbs, despite the exposure of the epitopes recognized by these antibodies on two representative native viruses (Du156.12 and JRFL), suggesting that the affinity of mAb might equally be crucial for neutralization, as the epitope recognition.

**Conclusions:**

Our study suggests that the anti-V3 antibodies derived from subtype-C infected Indian patients display neutralization potential against tier 1 viruses while such activity may be limited against more resistant tier 2 viruses. Defining the fine epitope specificities of these mAbs and further experimental manipulations will be helpful in identification of epitopes, unique to clade C or shared with non-clade C viruses, in context of V3 region.

## Background

The non-covalently associated surface (gp120) and transmembrane (gp41) subunits of the envelope glycoprotein are decorated on the surface of Human Immunodeficiency Virus Type-1 (HIV-1) as a trimeric spike [1], and serve as a target for broadly neutralizing monoclonal antibodies (bNAbs) [2-4]. Because of its involvement in the primary steps of receptor [5] and co-receptor binding [6], the envelope gp120 has been identified as a major target for HIV-1 NAbs [2,7-10]. However, the antigenic variability of exposed regions and low immunogenicity of conserved domains on gp120 impose great challenges to identify the vulnerable targets on HIV-1 [2,4,11]. Nevertheless, the conserved epitopes on gp120 have been identified using antibodies from neutralizing sera [12-14] and bNAbs [9,10,15-17], which include the antibodies directed to the CD4 receptor binding site (CD4bs) and co-receptor binding site mainly the third variable region (V3) [10,18-22].

The crystal structure of V3 resolved recently shows that V3 protrudes ~30 Å from the CD4-bound gp120 core, and this extended structure can be divided into three regions: the base (residues 1–8 and 25–35), the stem (9–14 and 18–24) and the crown (residues 15–17) (residue numbering w.r.t. V3) [23,24]. The V3 loop of gp120 is highly antigenic in humans [25-28], and was previously recognized as the principal neutralizing domain of HIV-1 [29]. However its role was shown to be restricted to type specific viruses [30,31] and such an observation was supported by the extensive sequence variation in V3 from different viral isolates [32,33]. Given the critical interaction with the co-receptors (CXCR4 or CCR5) on host cells, V3 conventionally must retain structurally conserved elements required for binding [34,35]. More recently, studies have revealed that the V3 domain possesses conserved structural motifs despite the sequence variation, and is often accessible on the virus surface as a target for bNAbs [21,36-38].

Although, the V3 loop displays high structural conservation, yet the degree of cross reactive anti-V3 antibody response in individuals infected with diverse HIV-1 subtypes varies substantially [39]. This difference in antibody response to V3 loop, has been shown to be primarily determined by the four amino residues in the V3 crown (GPGQ or GPGR), which mostly form a type II β-turn [19,40]. Interestingly, the anti-V3 monoclonal antibodies (mAbs) isolated from non-clade B infected individuals, bearing GPGQ at the tip of V3 display better neutralization capacity than subtype-B (having GPGR) derived anti-V3 mAbs [19]. Such an observation was substantiated by a study showing a high neutralization potential of the anti-V3 mAbs derived from Cameroon subjects infected with viruses harboring GPGQ (subtype-AG) at the V3 crown [21]. Further, in an immunization study carried out in rabbits with a gp120 DNA prime followed by a boost with various cholera toxin B (CTB) fusion proteins containing the V3 sequences from different HIV-1 subtypes, the CTB fusion protein containing a consensus-C (con-C) V3 sequence (V3C-CTB) (having a GPGQ motif at V3 crown), elicited a highly potent HIV-1 neutralizing response compared to the other V3-CTB constructs [41].

A limited number of mAbs have been generated so far against the HIV-1 subtype-C viruses including mAbs against the V3 loop despite the fact that subtype-C accounts for more than 50% of the global HIV-1 infections [42]. The only known human anti-V3 bNAb is from a clade B infected patient [15]. Keeping in view the above facts, we generated here three anti-V3 human mAbs from HIV-1 infected Indian patients, using the EBV immortalization method of human hybridoma technology. The functional analysis of the HIV-1 antibodies generated in this study revealed cross reactive binding and neutralization of the viruses tested.

## Results

### Characteristics of the anti-V3 mAbs generated from HIV-1 infected donors

A total of 3321 culture wells (96 well plate) of PBMCs derived from 33 HIV-1 infected patients were established. After 2–3 weeks, the culture supernatants of approximately 6% transformed wells tested positive for reactivity with V3C-CTB (Table 1). Three heterohybridomas producing anti-V3 mAbs were generated from different individuals (IDs; 277, 903 and 904). The mAbs belong to IgG1 subclass with one (904) using a lambda and the other two (277 and 903) with kappa light chain genes. Interestingly, the kappa light chain Abs displayed the same immunoglobulin heavy chain variable (IGHV) gene usage (3-30*03) while the other Ab (904) showed 1-8*01 gene usage (Table 2). The complementarity determining region 3 for the heavy chain (CDRH3) were different for each mAb indicating their uniqueness (Table 2).

**Table 1:** Characteristics of the screening of EBV transformed B-cell cultures with V3C-CTB

^**1**^**Number of HIV-1 infected patients**	**33**
^**2**^**Total number of PBMCs isolated (million)**	321.2
^**3**^**Number of wells plated**	3321
^**4**^**Number of wells positive for V3-CTB**	199
^**5**^**Number of anti-V3 antibody secreting cell lines**	3

^1^33 HIV-1 infected drug naïve patient donor samples were recruited for antibody production.

^2^A total of 321.2 million PBMCs were isolated from these 33 patients with a mean of 9.72 million per individual (range = 1.7 to 30 million).

^3^The PBMCs were plated in 96 well tissue culture plates @ 80-100 k cells/well and approximately 6% (199/3321) of the wells were positive with V3-CTB^4^.

^5^Three anti-V3 monoclonal antibody secreting clones 277, 903 and 904 were stabilized.

**Table 2:** Immunogenetic analysis of human anti-V3 monoclonal antibodies

**#**	^**1**^**mAb**	**Specificity**	**Isotype**	^**2**^**IGHV**	^**3**^**CDRH3**
**1**	277	V3 (gp120)	IgG1 κ	3-30*03	ATLPVVTPATEPFDF
**2**	903	V3 (gp120)	IgG1 κ	3-30*03	AKHYAEGGLDV
**3**	904	V3 (gp120)	IgG1 λ	1-8*01	ARFALQSYIVSTDSYDIDY

^1^Three anti-V3 monoclonal Abs listed were derived from HIV-1 infected Indian donors.

^2^Immunoglobulin gene usage for heavy (IGHV) and amino acid sequences of ^3^CDRH3 domains was determined using IMGT system; an asterisk indicates allele.

### Cross reactive binding and epitope mapping of anti-V3 mAbs by ELISA

**Figure 1:**
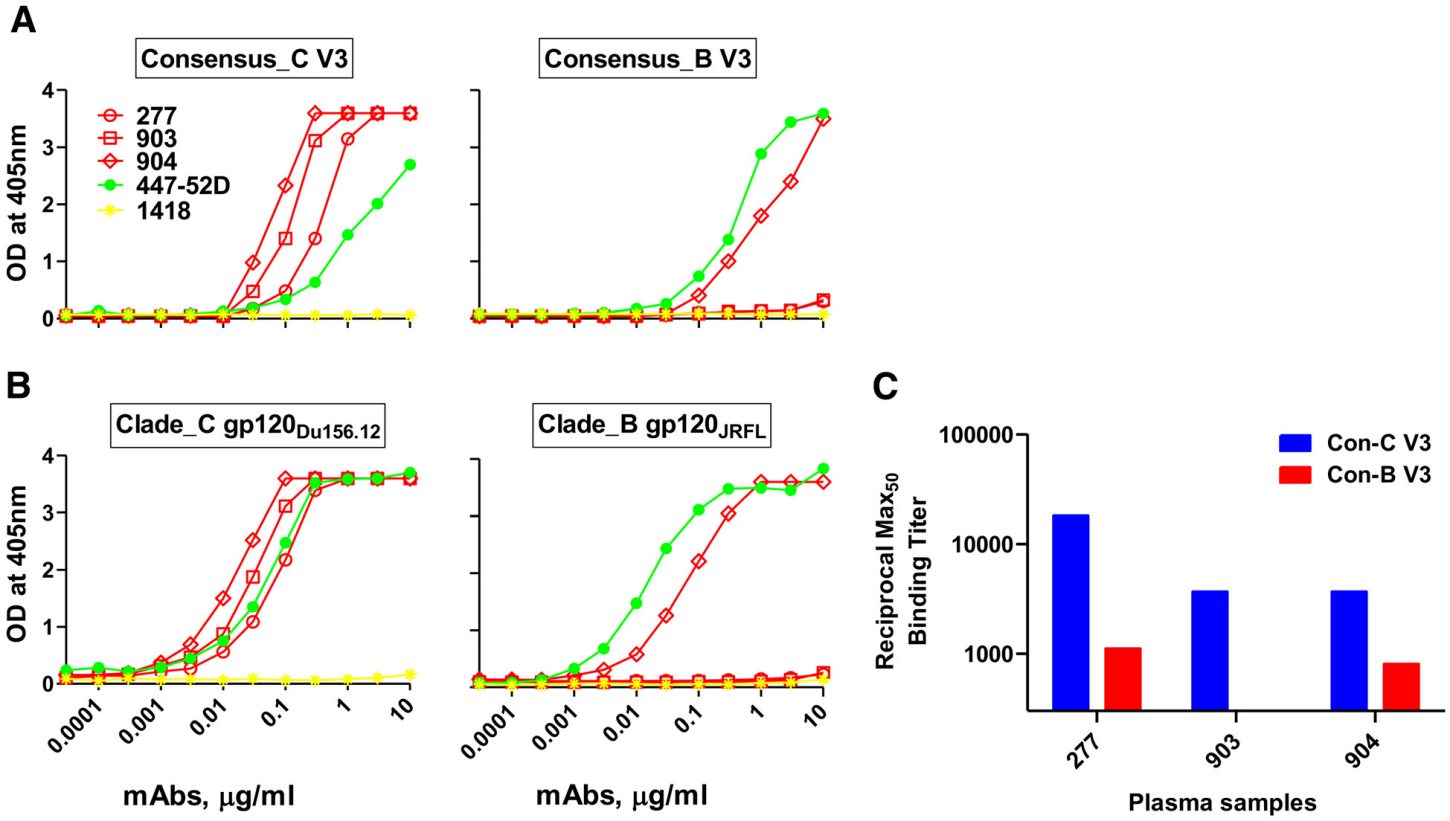
**Relative binding affinity of anti-V3 antibodies to HIV-1 derived peptides and proteins.** The binding pattern of anti-V3 mAbs derived from Indian donors (red) and 447-52D (an anti-V3 Ab isolated from HIV-1 subtype-B infected American individual) (green) to consensus-C and B V3 peptides (**1A**), and subtype-C (Du156.12) and subtype-B (JRFL) derived envelope gp120 proteins (**1B**). The binding of anti-V3 mAbs was tested by ELISA using mAbs at a concentration ranging from 10 to 0.00003 μg/ml (12 dilutions). Human anti-parvovirus B19 mAb 1418 was used as negative control. Relative reactivity of anti-V3 plasma antibodies to consensus-B and C V3 peptides is shown in terms of 50% ELISA binding titers (Max50) for three patients from whom antibodies were generated (**1C**). Two plasma samples 277 and 904 showed cross clade reactive binding while 903 displayed subtype-C specific binding.

All the plasma samples from patients recruited for this study, were previously screened for their relative binding to V3C and V3B peptides (Andrabi et al., submitted) and the data is here shown for only three samples from which anti-V3 mAbs were isolated (Figure 1C). The peptide binding assays revealed the cross reactive binding potential of the anti-V3 Abs in the plasma sample 277 and 904 while 903 showed subtype-C V3 specific binding.

In order to determine the specificity of the anti-V3 mAbs generated from these three patients, we performed the ELISA binding titration of the anti-V3 mAbs with recombinant envelope gp120 proteins and HIV-1 derived peptides (Table 3). In addition to three Indian anti-V3 mAbs, which were generated in this study, we also tested the binding of an anti-V3 bNAb (447-52D), isolated from a subtype-B HIV-1 infected individual living in USA. The binding curves of mAbs to con-C and B V3 peptides and subtype-C (Du156.12) and B (JRFL) gp120 proteins are depicted in the representative Figure 1. The mAbs were also tested with a series of peptides and proteins for quality control purpose and the 50% maximal binding (Max50) binding titers are summarized (Table 3). Overall, the mAbs 277 and 903 showed binding to subtype-A or C V3 while the antibody 904 also reacted with subtype-B V3 region (Figure 1A-B, Table 3). We further tested the mAbs by ELISA binding assays using linear overlapping peptides encompassing mainly V3 region flanked with second constant (C2) and third constant (C3) region of gp120, to identify the core epitope recognized and found that all these anti-V3 mAbs, including mAb 447-52D, bind to the crown of V3 loop (Table 4).

**Table 3:** Binding of mAbs to subtype-A, B and C derived HIV-1 proteins and peptides

		^**2**^**anti-V3 mAbs**				
^**1**^**Protein/peptide**	**Subtype**	^**I**^**277**	^**I**^**903**	^**I**^**904**	^**A**^**447-52D**	**1418**
**Con-C V3**	C	*0.33*	*0.109*	**0.046**	*1.2*	>10
**Con-B V3**	B	>10	>10	*0.623*	*0.323*	>10
**Du156.12**	C	**0.047**	**0.019**	**0.009**	**0.035**	>10
**JRFL**	B	>10	>10	**0.04**	**0.01**	>10
**92RW020**	A	**0.098**	**0.05**	**0.028**	*0.323*	>10
**SF162**	B	>10	>10	>10	**0.004**	>10
**MN**	B	>10	>10	*2.27*	**0.011**	>10
**MPER**	C	>10	>10	>10	>10	>10
**IDR**	C	>10	>10	>10	>10	>10
**p24**	B	>10	>10	>10	>10	>10
**BSA**	NA	>10	>10	>10	>10	>10
**PP**	NA	>10	>10	>10	>10	>10

^1^List of recombinant proteins and peptides derived from subtype-A, B and C HIV-1 viruses, NA: Not applicable.

^2^Four anti-V3 antibodies, three from Indian^I^ (277, 903 and 904) and one from American^A^ (447-52D) HIV-1 infected donor were tested for their relative binding. The binding activity of anti-V3 mAbs against proteins and peptides was tested by ELISA using mAbs at a concentration ranging from 10 to 0.00003 μg/ml (12 dilutions). The 50% binding titers (Max50, conc. μg/ml) of each antibody against the corresponding protein or peptide is depicted as numerical values in Bold (high affinity), Italic (low affinity) and >10, where Max50 value was not reached. Human anti-parvovirus B19 mAb 1418 was used as negative control. Each experiment was performed at least two independent times.

**Table 4:** Epitope mapping of anti-V3 mAbs with consensus-C V3 overlapping peptides

	^**1**^**Amino acid sequence of C2-V3-C3 overlapping peptides**	^**2**^**anti-V3 mAbs**				
**Peptide ID**	**IIVHLNESVEIVCTRPNNNTRKSIRIGPGQTFYATGDIIGDIRQAHCNISEEKWNKTLQ**	**277**	**903**	**904**	**447-52D**	**1418**
**9256**	**IIVHLNESVEIVCTR**	>10	>10	>10	>10	>10
**9257**	**LNESVEIVCTRPNNN**	>10	>10	>10	>10	>10
**9258**	**VEIVCTRPNNNTRKS**	>10	>10	>10	>10	>10
**9259**	**CTRPNNNTRKSIRIG**	>10	>10	>10	>10	>10
**9260**	**NNNTRKSIRIGPGQT**	>10	>10	>10	**0.049**	>10
**9261**	**RKSIRIGPGQTFYAT**	*4.04*	*1.37*	*0.327*	*0.149*	>10
**9262**	**RIGPGQTFYATGDII**	>10	>10	>10	>10	>10
**9263**	**GQTFYATGDIIGDIR**	>10	>10	>10	>10	>10
**9264**	**YATGDIIGDIRQAHC**	>10	>10	>10	>10	>10
**9265**	**DIIGDIRQAHCNISE**	>10	>10	>10	>10	>10
**9266**	**DIRQAHCNISEEKWN**	>10	>10	>10	>10	>10
**9267**	**AHCNISEEKWNKTLQ**	>10	>10	>10	>10	>10

^1^Amino acid sequences of linear overlapping peptides encompassing the third variable region (V3: Underlined (middle)), flanked by second (C2: (left)) and third (C3: (right)) constant regions and are aligned with the corresponding consensus-C gp120 sequence.

^2^Four anti-V3 antibodies, three from Indian^I^ (277, 903 and 904) and one from American^A^ (447-52D) HIV-1 infected donor were tested for their binding to overlapping peptides by ELISA using mAbs at a concentration ranging from 10 to 0.00003 μg/ml (12 dilutions).

The 50% binding titers (Max50, conc. μg/ml) of each antibody against the corresponding peptides is depicted as numerical values in Bold (high affinity), Italic (low affinity) and >10, where Max50 value was not reached. Human anti-parvovirus B19 mAb 1418 was used as negative control. Each experiment was performed at least two independent times.

### Binding of anti-V3 mAbs to HIV-1 native, intact virions

**Figure 2:**
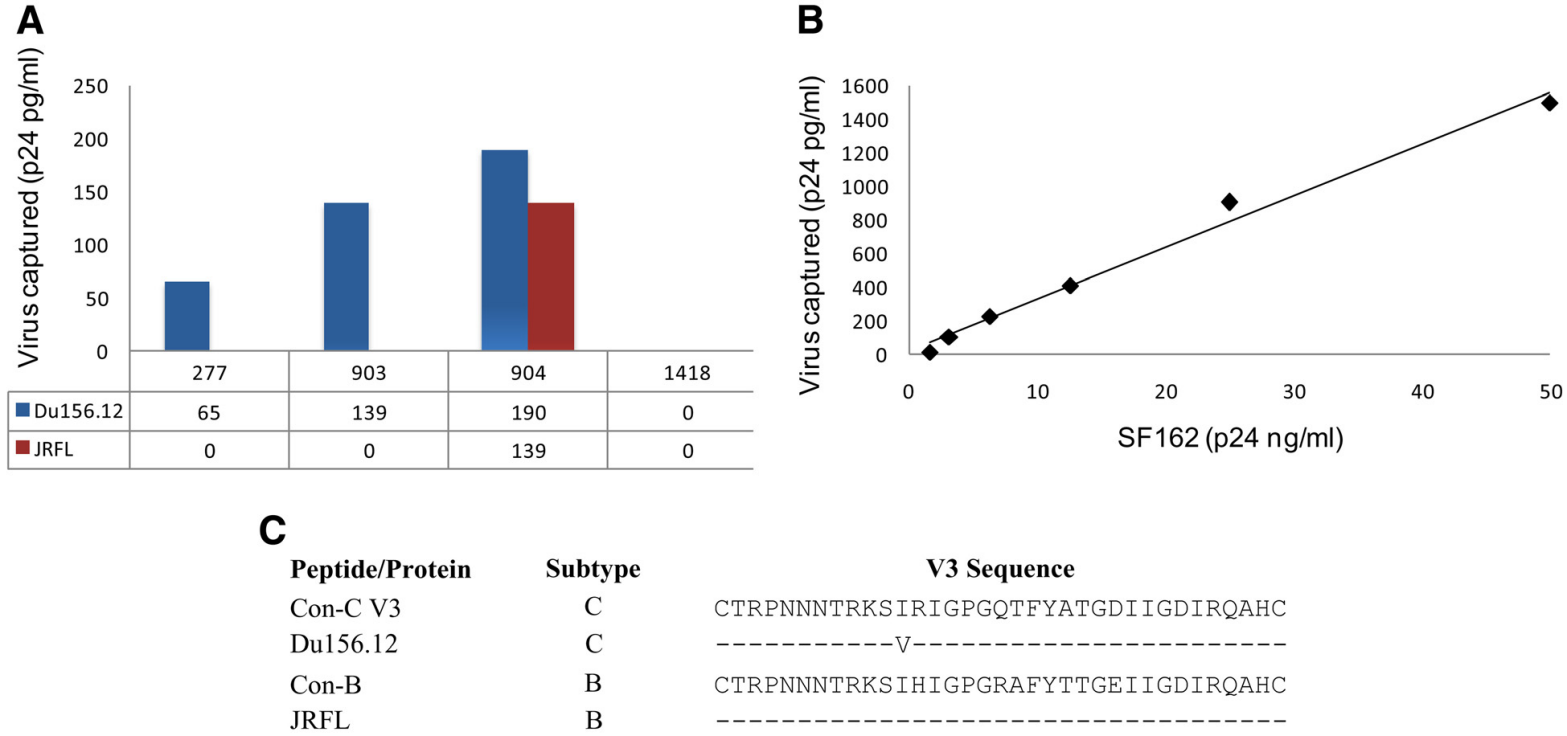
**Binding of anti-V3 mAbs to native viruses.** Binding of anti-V3 MAbs to native, intact HIV-1 virions, Du156.12 (subtype-C) and JRFL (subtype-B). The supernatants of the two viruses were used at a final p24 concentration of 25 ng/ml. Antibody 904 displayed binding to both viruses (Du156.12 and JRFL), while antibodies 277 and 903 showed subtype-C (Du156.12) specific binding (**A**). The unrelated mAb 1418 against parvovirus B19 served as a negative control.The anti-V3 mAb 447-52D (known to bind SF162, a clade-B virus) and SF162 were used to validate the experiment. The binding of 447-52D to intact virus was performed by using a fixed concentration of antibody (10 μg/ml) with two-fold dilution of SF162 virus starting with 50 ng/ml of virus (**B**). The virus capture is determined by measuring the level of p24 (picograms per milliliter) released when bound virus is lysed with detergent. The viruses (Du156.12 and JRFL) were chosen for intact virion binding assay on the basis of their resemblance to the consensus-B and C V3 loop sequences (**C**). The V3 sequence of these viruses is aligned with their corresponding consensus-C and B V3 loop sequence using Seqpublish program (http://hiv.lanl.gov).

The binding of Abs to HIV-1 derived peptides or proteins does not necessarily mean that these mAbs will be able to bind intact viruses, which essentially present a more native conformation. In order to address this possibility, we tested the anti-V3 mAbs for binding with Du156.12 (subtype-C) and JRFL (subtype-B) viruses in an intact virion binding assay. The two viruses (Du156.12 and JRFL) were chosen for this experiment on the basis of their V3 sequence similarity with the corresponding con-C and B V3 sequence (Figure 2C). Consistent with the binding of anti-V3 mAbs to Du156.12 and JRFL derived envelope gp120 proteins, the three mAbs showed differential binding affinity to intact virions, 904 displaying high affinity as compared with 277 and 903. In addition, the mAb 904 retained the cross-reactive binding potential to the intact viruses, evident from the binding pattern (Figure 2A). The experiment was validated by testing the intact virion binding of anti-V3 mAb 447-52D to SF162 virus, known to have a well exposed V3 region and allows accessibility to Ab without any interference [43-45]. The binding of 447-52D to SF162 intact virus revealed a very high binding affinity (more than five folds) of mAb 447-52D as compared to binding of three anti-V3 mAbs to Du156.12 and JRFL at equivalent viral concentration (i,e 25 ng/ml) (Figure 2A-B). This differential binding of mAbs to intact virions could also be attributed to the number of potential N-glycosylation sites (PNGS) within the V1/V2 region (3 for SF162, while 6 and 7 PNGS for Du156.12 and JRFL respectively) of these viruses [46]. Overall the results suggest that the V3 epitopes recognized by three anti-V3 mAbs were exposed on both Du156.12 and JRFL, however mAbs 277 and 903 were not able to bind JRFL due to their subtype-C specific binding activity.

### Neutralization potential of anti-V3 Abs against HIV-1 subtype-A, B and C viruses

The anti-V3 mAbs were tested to assess their capacity to neutralize a panel of eleven tier 1 and 2 viruses from different HIV-1 subtypes using the standard TZM-bl cell assay. The anti-V3 mAbs showed effective neutralization against two, a subtype-A (DJ263) and a subtype-C (MW965), out of five tier 1 viruses while only one virus (a subtype-C, HIV-001428) out of six tier 2 viruses was neutralized by two of the mAbs (903 and 904) with relatively low efficiency (Table 5). None of the Indian anti-V3 mAbs were able to neutralize any of the subtype-B viruses, despite antibody 904 showing cross reactive binding, nevertheless, the number of viruses tested here was limited. In contrast, the mAb 447-52D reached IC50 neutralization titers with 5/11 viruses tested, consistent with the previous studies [15,19].

**Table 5:** Neutralization of HIV-1 viruses by anti-V3 mAbs in TZM-bl assay

				**anti-V3 mAbs**				
**#**	**Virus ID**	**Tier**	**Clade**	**277**	**903**	**904**	**447-52D**	**1418**
1	**MW965**	1	C	**0.34**	**0.15**	**<0.1**	**<0.1**	>30
2	**DJ263**	1	A	*8.2*	*1.7*	**0.4**	**<0.1**	>30
3	**SF162**	1	B	>30	>30	>30	**<0.1**	>30
4	**JR-CSF**	1	B	>30	>30	>30	**0.3**	>30
5	**92RW009**	1	A	>30	>30	>30	>30	>30
6	**HIV-001428**	2	C	>30	*15.4*	*22.8*	>30	>30
7	**ZM109F.PB4**	2	C	>30	>30	>30	>30	>30
8	**ZM233M.PB6**	2	C	>30	>30	>30	>30	>30
9	**Du156.12**	2	C	>30	>30	>30	>30	>30
10	**JRFL**	2	B	>30	>30	>30	20	>30
11	**RHPA4259.7**	2	B	>30	>30	>30	>30	>30

The cross neutralizing activities of mAbs generated from Indian patients along with 447-52D (also an anti-V3 antibody), were evaluated against the tier 1 and 2 subtype_A, B, C viruses indicated on the left. The antibodies are shown with antibody IDs (numerical values in bold) at the top of each column. Human anti-parvovirus B19 mAb 1418 was used as negative control. The numerical values in the boxes below are the 50% neutralization titers (IC50) defined as the concentration (μg/ml) of mAb which neutralized 50% of viral infection in the assay. For clarity, this information is coded: IC50 < 1 μg/ml; (Bold), IC50 > 1 μg/ml; (Italic) and >30, where IC50 was not reached. Each experiment was performed at least two independent times.

## Discussion

Human monoclonal antibodies against the HIV-1 envelope glycoproteins are useful tools in the structural and functional analysis of the viral envelope and have crucial roles in guiding the design of prophylactic anti-HIV vaccines. Despite a huge expansion of HIV-1 subtype-C worldwide, the clade-C viruses remain to be one of the least studied subtypes especially in terms of HIV-1 neutralizing antibodies. Using the rationale from previous studies showing that viruses with GPGQ residues at the tip of the V3 crown of the HIV-1 envelope induce potent and cross reactive NAbs as compared to viruses with GPGR motif, we generated here three anti-V3 mAbs from Indian donors presumably infected with subtype-C viruses bearing GPGQ at the V3 crown [42]. The functional analysis of the Abs generated reveals a potent neutralization potential with tier 1 viruses while such activity was limited with the tier 2 viruses tested.

The anti-V3 Abs were selected from EBV-transformed B-cell cultures of 33 HIV-1 infected antiretroviral drug naïve patients using V3C-CTB fusion protein [47]. The advantage of using a conformationally constrained instead of a linear V3 peptide for selection of mAbs from cultures or as animal immunogens has been previously demonstrated [36,41,48-50]. We found 1-25% (mean = 6%) of the transformed wells positive for binding with V3C-CTB in the first screening. The characteristic nature of the B-cells from HIV-1 infected subjects and the conditions used to immortalize them apparently affects the number and type of Ab-producing cell lines that grow out [51]. The overall positive percentage of Ab secreting culture wells was relatively good and could be attributed to the high titers of anti-V3 Ab reactivity of the corresponding plasma [28]. In contrast to a high percentage of positive secretors in the initial screening, we were able to stabilize only three (277, 903 and 904) anti-V3 Ab producing B-cell clones. This loss could be in part due to the outgrowth of the non-secretor B cells over the secretor B cells in subsequent steps of secondary screening, cell fusion and dilution cloning process. Moreover, the B cells from HIV-1 infected patients are mostly dysfunctional and polyclonally activated [52], and such properties have been associated with a low persistence of EBV infectivity [51,53,54].

The amino acid sequence variation of V3 across the various HIV-1 subtypes is often related to a differential immune response focused to V3 which is expected to originate due to the subtype specific conformational differences in the V3 region [55,56]. For instance, the HIV-1 V3 crown residues GPGQ in non-clade B and GPGR in clade B viruses respectively are the major determinants of Ab binding and neutralization [19,40]. Epitope mapping of the anti-V3 Abs with overlapping V3 peptides reveal that their core epitopes lie in the crown region only. Indeed, the recent immunological and structural studies of anti-V3 mAbs have observed similar pattern of binding, wherein essentially all the anti-V3 mAbs bind to ~14 residues in the V3 crown [57-59]. Two of our anti-V3 mAbs (277, 903) showed binding to subtype-A or C but not to subtype-B derived proteins and peptides while mAb 904 displayed cross reactive binding with subtype-B as well. The binding pattern (in context of clade specific or cross reactive V3 antibodies) of the two anti-V3 mAbs 903 and 904 was similar to binding of polyclonal anti-V3 plasma antibodies from the respective patients, however it was different for mAb 277 in the context of the plasma Abs of this patient (Figure 1A-C). The finding highlights the importance of pre-screening of plasma for binding to peptides from different viral subtypes prior to isolation of mAbs. One plausible reason for the non-binding of mAb 277 to the V3B peptide in contrast to its corresponding polyclonal plasma may be attributed to the higher number of subtype-C specific B-cell clones in the B cell repertoire of this patient, as indicated by its very high binding to V3C (Figure 1C). It may also partly be ascribed to biased selection with a CTB construct containing only V3C sequence, which might allow it to preferentially pick up the B cell clone with clade-C V3 specificity. Interestingly, the anti-V3 mAbs 277 and 903, which show clade-A or C (both having a common GPGQ crown motif) specific binding use the same variable heavy chain gene (VH3-30) whereas the cross reactive mAb 904 uses a different VH gene (Table 1). The finding suggests a possible association of antibody gene usage with epitope specificity, however the number of the mAbs generated in this study is too small for comparison. Remarkably, a recent analysis of anti-HIV Abs has pointed out a preferential usage of VH5-51 gene usage of anti-V3 Abs [60], and such a preference was later shown to define a conserved antigenic structure within the V3 [61].

The Ab accessibility on the HIV-1 native virus is often challenged by the glycosylation pattern and epitope masking [39,62,63]. This effect has been particularly recognized for the V3 region wherein the neighboring regions including V1/V2 shield the epitopes recognized by anti-V3 Abs [46]. Although studies have suggested that the HIV-1 V3 loop remains accessible on most of the viruses [36], however this information is limited to subtype-B viruses and remains to be explored for other subtypes. Consistent with the binding to gp120 proteins (Du156.12 and JRFL), the three anti-V3 mAbs were able to bind intact native virions with a similar binding pattern (Figure 2A). The results suggest that V3 epitopes are well exposed over the intact trimeric viruses (Du156.12 and JRFL), and these findings are highly supported by previous work in the literature [36,43]. The rationale of using same proteins (gp120) and its corresponding viruses (intact virion) for the binding assays was to minimize the effect of both, the V3 loop sequence and the neighboring regions, on the local and global orientation of V3 and on the subsequent binding of Abs.

The anti-V3 mAbs showed potent neutralizing activity against subtype-A and C tier 1 viruses, however this activity was restricted for tier 2, especially the subtype-B viruses. The finding was intriguing given the ability of the anti-V3 mAbs to bind to two representative intact virions of subtype-B (JRFL) and subtype-C (Du156.12), and yet failing to reach IC50 neutralization titers. However, it should be noted that mAbs 903 and 904 which display a better affinity than 277, showed neutralization of up to 19% at 30 μg/ml, though not reaching IC50 neutralization titers, with these viruses (data not shown). Overall, the data suggest that higher concentrations of these mAbs may be effective in neutralization, however that needs to be confirmed in detail. Together, these results suggest that effective concentrations for binding and neutralization may vary substantially, and the high affinity binding by mAb might be critical for neutralization. The data are highly supported by various studies conducted previously [15,36].

## Conclusions

We isolated here three anti-V3 mAbs from HIV-1 infected donors from India which can effectively neutralize tier 1 viruses but are less effective with tier 2 viruses, however this needs to be confirmed by testing them with a broader panel of viruses from different HIV-1 subtypes. This study demonstrates the importance of pre-screening of plasma Abs for cross reactive binding, for production of mAbs and the idea can likewise be employed for other antigenic regions. Also the study highlights the significance of the antibody affinity, which may probably be equally important as its epitope accessibility, for effective viral neutralization. Furthermore, the analysis of the mAbs generated in this study will allow us to identify epitopes that are unique to clade C viruses and also those that are shared with other subtypes in the context of V3 loop, and the data may provide useful information for HIV-1 vaccine design.

## Material and methods

### Ethics statement

The study was approved by the ethics committee of All India Institute of Medical Sciences (AIIMS) New Delhi, and the written informed consent for research and publication of the data was obtained from all the participants.

### Study subjects

Thirty-three HIV-1 seropositive drug naive patients (18 males and 15 females) within the age range of 20–57 years (median = 33 years) were recruited in this study from the Regional STD Teaching Training & Research centre, Safdarjang Hospital, New Delhi, India during the period 2008–2011. The patients had a median CD4 count of 449 (range = 203-966) cells/cubic millimeter (Additional file 1: Table S1). The whole blood samples of HIV-1 positive donors were collected in EDTA vacutainers, plasma was separated by centrifugation at 300 g and stored in aliquots at - 80°C until use. The plasma samples were heat inactivated at 56°C for 1 h before using in the assays. The patients have been previously shown to have high titers of anti-V3 Abs in their plasma and a good proportion of these V3 directed Abs displayed cross reactivity [28], (Andrabi et al., submitted). Presumably, the patients were infected with subtype-C viruses, which is a major subtype in India [64,65]. Indeed, the envelope sequences (partial C2-C5 of envelope gp120) of a few patients revealed that majority of the patients were infected with subtype-C viruses (Andrabi et al., submitted).

### Generation of anti-V3 human mAbs

The mAbs were generated using cellular techniques as previously described [66]. Briefly, peripheral blood mononuclear cells (PBMCs) were EBV transformed in 96-well plates and cultured with a polyclonal B cell activator, CpG [67], which enhanced EBV infection and B cell transformation. The wells containing Ab-producing cells were identified by testing the culture supernatants for binding activity to V3C-CTB [47]. Cells from wells that test positive were expanded and fused with the heteromyeloma cell line SHM-D33 (ATCC; catalog no. 1668). The fused cells that continued to make functional Abs were repeatedly cloned until monoclonality was achieved. The Abs were purified from culture supernatants using Protein G affinity columns (GE Healthcare) and concentration of the mAbs was determined by non-commercial ELISA. The anti-V3 mAb 447-52D generated previously from a clade B infected individual and a mAb 1418, specific to parvovirus B19 [68], were used as control Abs in this study.

### RT-PCR amplification of the Ig variable region of the heavy chain genes

Nucleotide sequence of Ig variable genes of human anti-V3 mAbs was determined as previously described [60,61]. The messenger RNA was extracted from hybridoma cell lines producing anti-V3 mAbs and reverse transcribed into cDNA using oligo dT primer. Amplification of the variable fragment was performed by PCR using different gene family-specific sets of primers and cDNA as template. Five forward primers, VH 1/5 [5^′^-CAG GTG CAG CTG GTG CAG TCT GG-3^′^, VH2 [5^′^-CAG GTC AAC TTA AGG GAG TCT GG-3^′^, VH3 [5^′^-GAG GTG CAG CTG GTG GAG TCT GG-3^′^, VH4 [5^′^-CAG GTG CAG CTG CAG GAG TCG GG-3^′^ and VH6 [5^′^-CAG GTA CAG CTG CAG CAG TCA GG-3^′^ were located at 5^′^ end of V genes. Reverse primer [5^′^-CTTGGTGGARGCTGARGAGACGGTGACC-3^′^ was located at the 3^′^ end of JH segment and up to 12 nucleotides at the 5^′^ of the constant region of IgG [69]. PCR amplification was performed using cycling program of 2 min at 94°C, 35 cycles of 60s at 94°C, 60s at 56°C, and 90s at 72°C, followed 8 min at 72°C. Ethidium bromide-stained 0.8% agarose gels were used to visualize the PCR products. The bands of the appropriate size were excised and cleaned with GeneElute Minus EtBr Spin Column (Sigma, USA). PCR products were sequenced (Macrogen, South Korea) in both directions using the primers applied for amplification. The sequence data were analyzed using the International ImMunoGeneTics (IMGT) information system (http://imgt.cines.fr).

### Envelope proteins and peptides

Five recombinant gp120s representing sequences of primary HIV-1 isolates from clade A, B and C (produced in 293 cells) and a p24 protein were purchased from Immune Technology Corp. (New York, NY). A set of 12 linear overlapping peptides (each 15mer with an 11 amino acid overlap or a 4 amino acid walk) corresponding to the sequence of consensus subtype-C V3 gp120 and CEF Control Peptide Pool (PP) (Cat. No. 9808) were obtained from the NIH AIDS Research and Reference Reagent Program (NIH, ARRRP). Two full length (35mer) peptides corresponding to the consensus-B (CTRPNNNTRKSIHIGPGRAFYTTGEIIGDIRQAHC) (V3B) and con-C (CTRPNNNTRKSIRIGPGQTFYATGDIIGDIRQAHC) (V3C) of V3 gp120, a 24mer con-C MPER (DLLALDSWKNLWNWFDITNWLWYIK) and a 19mer con-C IDR (LGIWGCSGKLICTTAVPWN) peptides of gp41 were selected from Los Alamos HIV-1 sequence database (http://hiv.lanl.gov), and were synthesized from Sigma Genosys, USA. The peptides were HPLC purified to >95% purity (based on information provided by company). The V3-cholera toxin B (CTB) fusion protein (V3C-CTB) and wild type CTB (WT-CTB) used for screening of antibody cultures were kindly provided by Prof. Susan Zolla Pazner from New York University School of Medicine.

### Binding assay

The binding activity of anti-V3 mAbs against gp120 proteins and peptides (including the V3 overlapping peptides) were tested by enzyme-linked immunosorbent assay (ELISA) as described [18]. Briefly, ELISA plates were coated overnight with protein or peptide at 1.0 μg/ml, blocked with 2% bovine serum albumin (BSA) in PBS, and then incubated with mAbs at a concentration ranging from 10 to 0.00003 μg/ml (12 dilutions). The bound mAbs were detected by incubation with alkaline phosphatase-conjugated goat anti-human IgG (γ specific) (SouthernBiotech, Birmingham, AL) followed by adding substrate to develop color and the plates were read at 405 nm. The relative affinities of mAbs were determined by measuring the concentration of mAbs required for 50% maximal binding (Max50), defined when the binding curve reached the saturation level as described [70].

The binding of mAbs to intact virions was determined with a capture assay as previously described [71]. Briefly, a 96-well plate was coated overnight at 4°C with goat anti-human immunoglobulin G (IgG) Fc Abs (Sigma) at 4.0 μg/ml, and then anti-V3 human mAbs at a saturating level of 10 μg/ml were added for 1.5 h incubation at 37°C. The plate was blocked with 0.5% BSA in phosphate-buffered saline (PBS) containing 10% goat serum and 10 μg of human IgG/ml. The culture supernatant, containing pseudotyped viruses (Du156.12 and JRFL) at a p24 concentration of 25 ng/ml, and the two fold diluted primary isolate (SF162) with a starting p24/ml concentration of 50 ng were added. The plate was incubated overnight at room temperature. Viruses captured by immobilized mAbs were lysed with 1% Triton-X in PBS. Between each step of the assay, the plate was washed with PBS containing 0.05% Tween 20, pH 7.4. The p24 in the virus lysate was quantified by using a commercial ELISA. The anti-V3 Ab 447-52D and SF162, a subtype-B virus known to be sensitive to most of anti-V3 Abs were used as assay control while the human mAb, 1418, was used as a negative control.

### Primary isolates and pseudoviruses

Eleven HIV-1 subtype A, B and C viruses including five primary isolates (MW965, DJ263, SF162, JR-CSF and 92RW009) and six pseudotyped viruses (HIV-001428, ZM109F.PB4, ZM233M.PB6, Du156.12, JRFL and RHPA4259.7) were used for this study. All the primary viruses and envelope clones were obtained from the NIH, ARRRP. The HIV-1 isolates were expanded by only one cycle of growth on phytohemagglutinin (PHA) and interleukin-2 (IL-2)-stimulated PBMCs, as described previously [45] to avoid alterations in *env* sequences due to multiple rounds of expansion. Pseudotyped viruses were produced by co-transfection of the rev/env expression plasmid and an env-deficient HIV-1 backbone vector (pSG3ΔEnv) into exponentially dividing 293 T cells (ATCC; catalog no. 11268), in 6-well tissue culture plates (Corning Inc) using calcium phosphate (Promega Inc) method. Pseudovirus-containing culture supernatants were harvested 48 hours post transfection filtered (0.45 μm pore size) and stored at −80°C in 1 ml aliquots. The 50% tissue culture infectious dose (TCID50) was determined in TZM-bl cells.

### Neutralization assays

Neutralization of viruses by anti-V3 mAbs was measured as a reduction in luciferase gene expression after a single round of infection of JC53bl-13 cells, also known as TZM-bl cells (NIH, ARRRP; catalog no. 8129), with viruses [72,73]. Briefly, 200 TCID50 of pseudovirus was pre-incubated for 1 hr at 37°C, 5% CO_2_ in 96-well flat-bottom culture plates, with serial dilutions of mAbs, starting from 30 μg/ml. Freshly trypsinized TZM-bl cells (10,000 cells in 100 μl of growth medium containing DEAE Dextran and protease inhibitor indianavir (in case of primary isolates only), were added to each well of the mAb/virus mixtures in duplicates. One set of control wells received cells plus pseudovirus (virus control) and another set received cells only (background control). After 48 hours of incubation at 37°C, 5% CO_2_, luciferase activity was measured by using the Bright-Glo Luciferase Assay System (Promega Inc.). The 50% inhibitory concentration of mAb (IC50) was determined at which relative luminescence units (RLU) were reduced 50% compared to virus control wells.

## Competing interests

The authors declare that they have no competing interests.

## Authors’ contributions

RA and KL designed the study, performed the data analysis and drafted the manuscript. RP and SS helped in study design. MB, AB and NW recruited all the HIV-1 infected patients. RA carried out majority of the experiments. RK, AN and PK helped in plasmid amplification for pseudotyped-virus generation, immunoglobulin variable gene sequence determination and dilution cloning experiments respectively. All authors have read and approved the final manuscript.

## Supplementary Material

Additional file 1 **Table S1.** Demographic and clinical data of 33 HIV-1 infected drug naive patients recruited for human monoclonal antibody production. Click here for file
