# Supplementary material for: Production and characterization of human anti-V3 monoclonal antibodies from the cells of HIV-1 infected Indian donors
Source: Virol J. 2012 Sep 12;9:196. doi: 10.1186/1743-422X-9-196 (PMC3493341; doi:10.1186/1743-422X-9-196)
Supplement: Additional file 1 — Table S1. Demographic and clinical data of 33 HIV-1 infected drug naive patients recruited for human monoclonal antibody production. [file 1743-422X-9-196-S1.doc]

**Table S1: Demographic and clinical data of 33 HIV-1 infected drug naive patients recruited for human monoclonal antibody production**

| **AIIMS ID** | **Time from First Diagnosis** | **Gender** | **Age** | **CD4 Count** |
| --- | --- | --- | --- | --- |
| **AIIMS223** | 2 | F | 20 | 450 |
| **AIIMS232** | 192 | M | 37 | 213 |
| **AIIMS239** | 15 | M | 25 | 366 |
| **AIIMS248** | 302 | F | 36 | 303 |
| **AIIMS249** | 395 | M | 32 | 312 |
| **AIIMS250** | 641 | F | 25 | 278 |
| **AIIMS253** | 26 | M | 22 | 436 |
| **AIIMS254** | 862 | M | 36 | 203 |
| **AIIMS255** | 1085 | M | 40 | 587 |
| **AIIMS264** | 623 | M | 35 | 442 |
| **AIIMS273** | 22 | F | 41 | 591 |
| **AIIMS275** | 2534 | M | 31 | 366 |
| **AIIMS277** | 282 | M | 37 | 235 |
| **AIIMS281** | 1142 | M | 40 | 401 |
| **AIIMS283** | 8 | F | 21 | 394 |
| **AIIMS285** | 1250 | F | 32 | 456 |
| **AIIMS289** | 3 | F | 26 | 229 |
| **AIIMS300** | 226 | F | 57 | 601 |
| **AIIMS901** | 1277 | M | 30 | 385 |
| **AIIMS902** | 2007 | F | 22 | 686 |
| **AIIMS903** | 2012 | M | 31 | 490 |
| **AIIMS904** | 1277 | F | 50 | 508 |
| **AIIMS905** | 2555 | M | 41 | 500 |
| **AIIMS906** | 1460 | M | 38 | 449 |
| **AIIMS907** | 1460 | F | 30 | 739 |
| **AIIMS908** | 912 | M | 33 | 462 |
| **AIIMS909** | 1642 | F | 26 | 380 |
| **AIIMS910** | 1460 | F | 26 | 440 |
| **AIIMS911** | 1170 | M | 44 | 648 |
| **AIIMS912** | 1095 | M | 32 | 926 |
| **AIIMS913** | 1080 | F | 35 | 966 |
| **AIIMS914** | 1460 | M | 35 | 679 |
| **AIIMS915** | 1320 | F | 42 | 759 |

^1^Days from 1^st^ Diagnosis: Number of days between 1^st^ day of HIV-1 detection and date of sample collection.

^2^Gender: Male (M), Female (F)

^3^Age: HIV-1 infected donors within age range of 18-60 years were included in the study

^4^CD4 count: Number of CD4+ T-cells/cubic millimetre of blood
